# Nanomechanical Optical Fiber with Embedded Electrodes Actuated by Joule Heating

**DOI:** 10.3390/ma7085591

**Published:** 2014-07-31

**Authors:** Zhenggang Lian, Martha Segura, Nina Podoliak, Xian Feng, Nicholas White, Peter Horak

**Affiliations:** Optoelectronics Research Centre, University of Southampton, Southampton SO17 1BJ, UK; E-Mails: lianzhenggang@gmail.com (Z.L.); N.Podoliak@soton.ac.uk (N.P.); xif@orc.soton.ac.uk (X.F.); nmw@orc.soton.ac.uk (N.W.); peh@orc.soton.ac.uk (P.H.)

**Keywords:** micro-structured optical fibers, optical fiber sensors, all-optical fiber devices, optical switching

## Abstract

Nanomechanical optical fibers with metal electrodes embedded in the jacket were fabricated by a multi-material co-draw technique. At the center of the fibers, two glass cores suspended by thin membranes and surrounded by air form a directional coupler that is highly temperature-dependent. We demonstrate optical switching between the two fiber cores by Joule heating of the electrodes with as little as 0.4 W electrical power, thereby demonstrating an electrically actuated all-fiber microelectromechanical system (MEMS). Simulations show that the main mechanism for optical switching is the transverse thermal expansion of the fiber structure.

## 1. Introduction

Microstructured optical fibers (MOF) have been designed in a large variety of geometries. Most common structures are based on hexagonally stacked geometries with solid cores, but various specialty fibers, e.g., suspended-core [1], suspended-web [2] or hollow-core fibers [3,4], have also been demonstrated. Many of these efforts were focused on control of dispersion, enhancement of the nonlinearity, reduction of the fiber transmission loss, or increase of sensitivity to environmental conditions in single-material MOFs [5]. Multi-material MOFs have also been developed for added functionality, e.g., hollow-core fibers filled with liquids [6] or gases [7] for sensing, with gases for high-power pulse compression [8,9] and nonlinear effects [10,11], with semiconductors for electronic functionality [12], and with integrated electrodes [13,14] and liquid crystals [15] for optical switching.

Optical fibers with optical properties that can be externally controlled after fabrication could open up a new range of applications, in particular in telecommunications, sensing, and imaging, following the successful model of chip-based microelectromechanical systems (MEMS) [16]. An example of such a reconfigurable fiber is the dual suspended-core nanomechanical optical fiber [17], which consists of two independent cores suspended by thin membranes and surrounded by air. The cores interact with each other via their evanescent optical fields; thus, mechanically changing the core-to-core separation modifies the optical coupling length and allows for active control of the propagating light. Various mechanisms have already been proposed to achieve this actuation. In [17], optical switching was demonstrated by pressurizing the gas-filled holes of the fiber. With just an 8-nm change of the core separation optical switching of the light at the fiber output from one core to the other was observed. Alternative mechanisms to induce optical switching that have been proposed for such fibers are electrostatic actuation [18] and optomechanical forces [2]. For many applications, forms of electrical actuation are preferred [12,13,14,15,18], but operation at low voltages/low powers would often only be achieved by placing electrically conducting electrodes close to the optically guiding cores, *i.e.*, by embedding metals into the fiber.

Here we report the fabrication of nanomechanical optical fibers with integrated electrodes by a multi-material co-draw technique: a dual suspended-core structure fabricated in lead-silicate glass (Schott F2) at the center of the fiber is surrounded by four tin electrodes. Using these fibers, we then demonstrate a novel electrically-induced actuation mechanism whereby electric current in the electrodes leads to Joule heating of the fiber by a few degrees Celsius, sufficient to modify the transmission properties of fibers of approximately 0.5 m length.

This design opens new possibilities for applications of optical fibers as MEMS [16]. Such MEMS-type fibers are transmitting light in the same way as standard fibers, but simultaneously they can perform some active signal processing functionality, such as switching or modulating, thereby avoiding e.g., the use of chip-based micromirrors and microlenses. Moreover, because of the long propagation length of fibers compared to integrated devices, already nanometer-scale actuation is sufficient to achieve observable effects [17,18].

## 2. Fabrication of Nanomechanical Optical Fibers with Embedded Metal Electrodes

**Figure 1:**
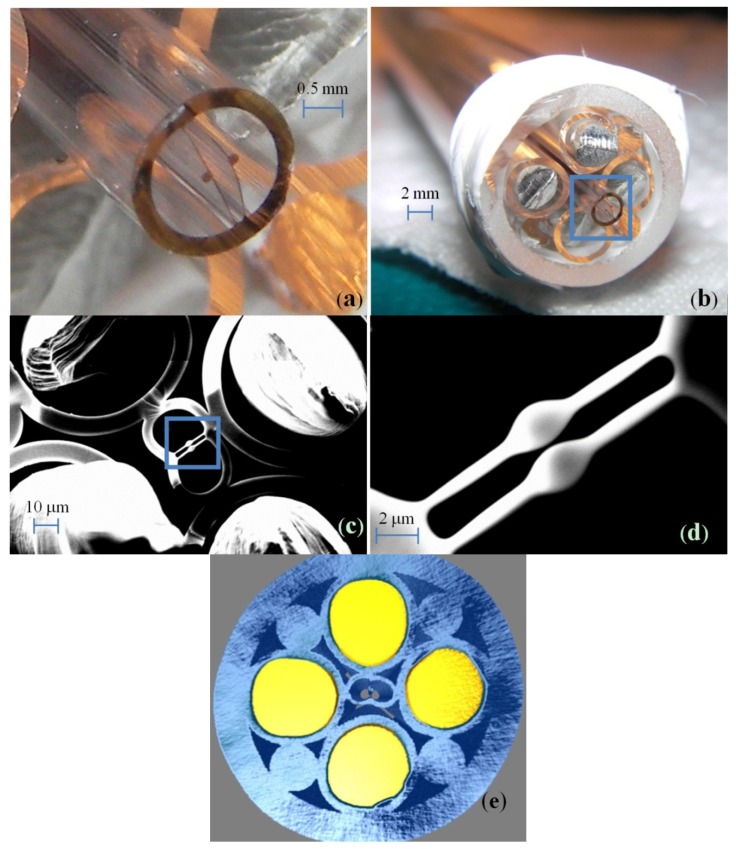
(**a**) Dual suspended-core cane; (**b**) Stacked preform with the cane in the center and four tin rods in glass tubes; (**c**) SEM picture of the dual-core fiber 1 with embedded metal electrodes after drawing. The length of the membranes is 12 μm, and the gap between the two cores is 200 nm; (**d**) Detail of the cores in the blue square of (**c**); (**e**) Advanced X-ray computed tomography image of the fiber, electrodes in yellow and glass in blue.

The electrode-embedded nanomechanical optical fiber was fabricated in several steps:

(i) A dual-core glass preform was fabricated in Schott F2 glass, which has a refractive index of *n*_0_ = 1.595 at an operating wavelength of 1550 nm leading to tighter mode confinement than silica [19]. Because of the low softening temperature of F2 glass (592 °C), glass extrusion with a stainless steel die could be used for preform fabrication, similar to the previous fabrication of other high-precision specialty fiber preforms based on compounds glasses including lead-silicate glasses [20], tellurite glasses [21] and chalcogenide glasses [22,23].
(ii) The preform was then drawn into a smaller dimension cane of 2 mm diameter; see Figure 1a.

(iii) In the next step, a multi-material preform was then assembled: The dual-core cane was placed in the middle of a F2 jacket tube (10/8 mm OD/ID) and surrounded by four glass tubes filled with tin rods (melting temperature 232 °C), as shown in Figure 1b. Four extra F2 rods were used to fill the gaps between the jacket and the tubes and to secure the position of the cane.
(iv) Under nitrogen purging conditions this preform was then pre-heated to a temperature above the melting temperature of tin for 60 min in order to melt the tin and condense it down to the bottom of the preform. This step was found to be critical to minimize air bubbles between the metal and the glass, thus avoiding discontinuities of the tin electrodes along the fiber.
(v) Finally, the preform was drawn into fiber using a drawing temperature of 730 °C [24]. Two different fibers from different preforms were fabricated with this method. Figure 1c shows a scanning electron microscopy (SEM) picture of one of the fibers with the two suspended cores clearly seen in the center; a magnified picture is shown in Figure 1d. We note that the fiber is not perfectly symmetric due to a small asymmetry of the stacked preform and slightly insufficient pressure being applied to the core structure during fiber drawing. Table 1 summarizes the dimensions of the two fibers.

**Table 1:** Dimensions of the fibers used for the optical experiments.

Fiber	Core size (μm)	Gap (μm)	Membrane thickness × Length (μm)
Fiber 1	2.0 × 3.1	0.2	0.5 × 12
Fiber 2	2.0 × 3.0	0.5	0.6 × 15

For both fibers the average diameter of the four tin electrodes is 50 μm. It was confirmed that the electrodes are continuous over tens of meters by measuring the electrical resistance along the fiber. Images produced by X-ray computed tomography also showed that the tin electrodes filled the glass tubes without air bubbles, see Figure 1e. The optical loss of fiber 1 was measured for TE and TM polarizations (parallel and orthogonal to the membranes, respectively) as 2.5 and 4.8 dB/m. The loss mechanisms are expected to be the intrinsic loss of lead-silicate glasses (1 dB/m at 1.55 μm) and scattering due to surface roughness. The latter affects the TM mode more since this mode has larger optical fields at the core surfaces, leading to larger losses than for the TE mode.

## 3. Optical Characterization

For the optical characterization of the fiber under electrical actuation, both ends of the fiber were carefully cleaved, removing the glass part but leaving the four electrodes for connecting purposes. The ends of the fiber were placed on two metallic V-grooves and fixed with magnets in such a way that the fiber was not bent or twisted along its length.

**Figure 2:**
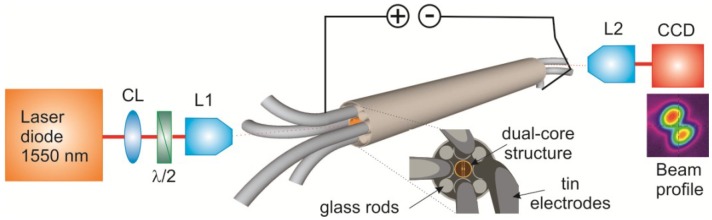
Setup for optical characterization of the dual-core fiber. CL: collimating lens; λ/2: half-wave plate; L1, L2: microscope objective lenses.

A diagram of the experimental setup for the optical characterization is shown in Figure 2. Linearly polarized light from a 1550 nm wavelength pigtailed laser diode (2 mW) was focused into one of the cores with a microscope objective (L1) with 40× (or 60×) magnification and numerical aperture NA = 0.65 (or 0.75). At the output, a 100× long working distance objective (L2) with NA = 0.85 was used to image the output beam onto a CCD camera (SCOR-20-1550, Ophir-Spiricon Inc., Logan, UT, USA).

The four electrodes at each end were connected to the V-groove using silver paint and two copper wires coming from the power supply were soldered to each V-groove. In this way, one side of the metal electrodes was connected to the positive side of a power supply, and the other side of the metal electrodes was connected to the negative.

## 3.1. Experiments with Fiber 1

We first performed experiments with a piece of fiber 1 in Table 1 of 65 cm length. When the voltage of the source was varied, the current showed a linear Ohmic response with a total resistance of 11 Ω. Light was coupled into the fiber using the 40× objective as lens L1 (Figure 2). The polarization state was chosen normal to the membranes (TM polarization) because of the slightly stronger core-to-core coupling and thus higher switching contrast of this mode compared to that of TE polarization.

**Figure 3:**
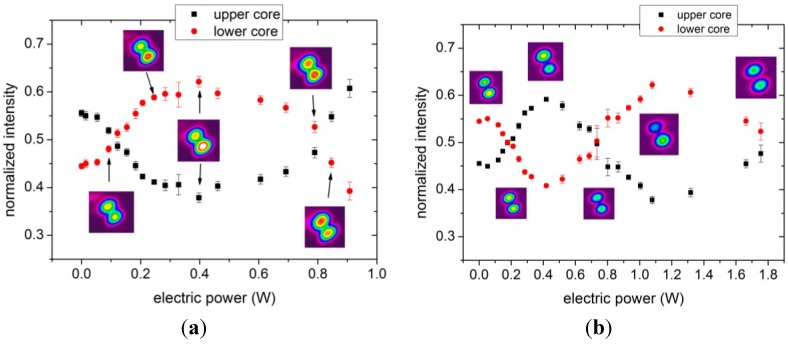
Normalized output intensity in the upper (black squares) and lower (red dots) cores as function of electric power for two different pieces of fiber 1 with fiber lengths of (**a**) 65 cm and (**b**) 70 cm. The intensity profiles shown were recorded with the CCD camera.

At room temperature, the guided light pattern shown on the CCD camera was stable. As the electric power increased and the fiber was therefore slowly heating up, a noticeable switching of light between the cores was observed, see Figure 3a. A sequence of light patterns was recorded consisting of 16 frames at 7.5 frames per second for each electric power level. The maximum intensity of light in each core was extracted from the 16 Gaussian profiles, averaged and normalized to the sum of intensities in both cores. The starting contrast was 0.55/0.45 (upper/lower core). As the electric power increased, a change in intensity of the light in each core was observed, see Figure 3a, reaching a maximum contrast of 0.38/0.62 at 0.4 W of electric power. The maximum applied electric power was 0.9 W where instabilities in the output light pattern and light coupling to higher order modes were observed. As shown in Figure 3a, an increase of power of 0.7 W was required to switch between the two points where both cores show the same output intensity. This behavior was repeatable, and the same results were observed when ramping the power up or down; no hysteresis effect was observed.

The experiment was repeated with a different section of fiber 1. This piece was 70 cm long with 13.4 Ω resistance and showed optical switching performance very similar to the previous piece of fiber. Again, the instability of the recorded output field pattern was observed at higher electrical powers (>0.6 W). However, we found experimentally that by decreasing the incident optical power four times with respect to the previous experiment (0.5 mW instead of 2 mW), the beam profile remained stable and we were able to increase the applied electric power up to 1.8 W, as can be seen in Figure 3b. The reason for this dependence of mode field stability on optical power is not exactly known at this time, yet we note that losses of 1 mW of optical power by material absorption in the fiber cores correspond to approximately 0.1 mW per μm^2^ of cross section, which is comparable to the electric heating power distributed over the much larger cross section of the electrodes. It is thus conceivable that laser light absorption, even at such low power levels, leads to small localized heating effects in the fiber cores which in turn modify the optical coupling length between cores and thus result in unstable, nonlinear back action on the light field distribution.

With this reduced optical power, the fiber showed continuous switching with electrical power up to 1.8 W with a maximum contrast of 0.38/0.62, Figure 3b. The switching shown in Figure 3b is not linear with power. The reason for this behavior is not fully understood, but it could be caused by a small temperature dependence of the launching conditions and/or by interference of higher order modes. However, both pieces of fiber 1 exhibited the same behavior from 0 to 0.9 W indicating that the results are repeatable with different fibers of similar dimensions.

## 3.2. Experiments with Fiber 2

As discussed in more detail in Section 4, we explain the observed optical switching by the thermal expansion of the fiber structure due to Joule heating by the electric current. In order to confirm this thermal mechanism, we carried out another set of experiments to compare the optical switching achieved by electric current applied to the electrodes and by external heating of the fiber. Here we used a 68-cm length of fiber 2 with a total resistance of 14 Ω and the 60× objective was used as lens L1 to couple TM-polarized light into the fiber.

A cylindrical heater was designed such that a fiber placed at the axis of the heater could be uniformly heated. The heater is 45 cm long and 8 mm in diameter, and it can be controlled in steps of 0.1 °C up to a maximum of 50 °C. A thermocouple measures the temperature in the middle of the heater. The two experiments, heating the fiber via the electrodes and externally, were carried out one after the other with the fiber placed inside the heater. First, the fiber was heated by applying electric current and then, after cooling, the temperature of the heater was increased.

**Figure 4:**
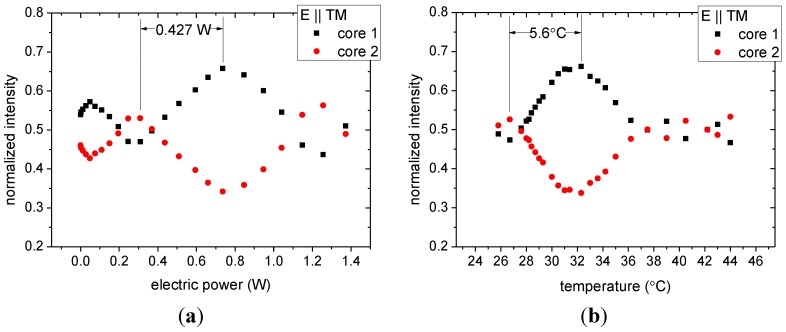
Normalized output intensity in core 1 (black squares) and core 2 (red dots) of fiber 2 as a function of (**a**) electric power; and (**b**) temperature.

The resulting output intensities from the two cores for the current heating are shown in Figure 4a. As can be seen in Figure 4a, the starting output contrast is nearly 0.50/0.50. The initial temperature for this experiment was 22.5 °C. At low electric power levels (<0.4 W) small changes of the intensity in the two cores are recorded, which increase to the largest observed contrast of 0.65/0.35 at 0.7 W electric power. The electric power needed to switch from one core to the other is 0.427 W.

The measurement results for switching by external heating are shown in Figure 4b. The room temperature had increased to 25.8 °C compared to the previous experiment, leading to a shift of the curves in Figure 4b with respect to those of Figure 4a, but otherwise the behavior observed in the two experiments is very similar. The largest contrast was found at 32 °C with external heating, *i.e.*, a temperature change of 5.6 °C was needed to switch light between the two fiber cores. The equivalence of the two experimental results confirms that the change in temperature is in fact the switching mechanism for the electrically driven actuation of our dual-core nanomechanical fiber. It also provides an indirect measurement of the fiber core temperature increase with Joule heating in the electrodes (5.6 °C for 0.427 W).

## 4. Modelling of Thermally Induced Switching 

The mechanism of optical switching in the dual-core fiber can be interpreted as follows. Being closely placed to each other, the optical cores are coupled through their evanescent fields, forming symmetric and antisymmetric superposition mode pairs for each polarization (TE and TM supermodes). Each of these modes is characterized by an effective mode index (*n_s_* and *n_a_* denote symmetric and antisymmetric supermode index, respectively), which depends on the optical coupling strength between the two cores. If light is launched into a single core, both supermodes are excited and an interference pattern is formed along the length of the fiber, which is observed as light periodically switching between the cores. The beat length, *L_b_*, the propagation distance at which the light transfers from one core to the other and back, depends on the mode index difference and the wavelength λ as *L_b_* = λ/(*n_s_* − *n_a_*). The fiber output thus depends on the total phase difference occurring between the supermodes along the fiber:

(1) $$\text{δ}=2\text{π}(n_{s}-n_{a})L/\text{λ}$$

where *L* is the fiber length. Modifying the fiber properties by some form of actuation leads to a change of δ, *i.e.*, a shift of the interference pattern of the supermodes that is seen as a continuous switching of the light between the cores at the output. One switch (half of a duty cycle) corresponds to a phase difference variation of π.

By running an electric current through the electrodes, Joule heating raises the temperature of the fiber. Unlike the previous study where switching was induced by optical core deformations caused by the metallic electrode expansion [13], in our fiber the influence of the electrode expansion is suppressed by the air gaps between the electrodes and the optical cores. The electrodes rather act as an internal heater. Having four electrodes connected to the power supply ensures uniform temperature of the internal part of the fiber eliminating stress due to temperature gradient. Thus, we identify three other contributions to the variation of δ: A. Thermal elongation of the fiber leads to a larger propagation distance and hence increases the phase difference (assuming that the mode indices do not change); B. The increase of the material refractive index with temperature leads to stronger localization of the optical fields inside the cores and thus reduces core-to-core coupling, which in turn decreases the phase difference between the supermodes; C. Uniform thermal expansion in the transverse direction of the inner fiber structure increases both the core size and the air gap between the cores, again reducing the core-to-core coupling efficiency. As a result, the change of the phase difference *d*δ between the supermodes induced by a temperature variation *dT* can be summarized as:

(2) $$d\text{δ}=2\pi L\left(\text{α}\left(n_{s}-n_{a}\right)dT+{\frac{\partial (n_{s}-n_{a})}{\partial T}|}_{\begin{array}{c} \text{material}\\ \text{index} \end{array}}dT+{\frac{\partial (n_{s}-n_{a})}{\partial T}|}_{\begin{array}{c} \text{transverse}\\ \text{expansion} \end{array}}dT\right)/\text{λ}$$

where α is the thermal expansion coefficient. The first term is positive, while the other two contribute with negative sign.

To quantify the impact of these three effects in the dual-core fiber, we calculated effective mode indices for the fundamental symmetric and antisymmetric modes and variation of the mode indices induced by the effects A, B and C using a finite element method (Comsol Multiphysics [25]). The optical and thermal properties of F2 glass used in the calculations (refractive index *n* = 1.595 at 1.55 μm, thermal expansion coefficient α = 9.2 × 10^−6^ K^−1^, and thermo-optic coefficient *dn*/*dT* = 2.7 × 10^−6^ K^−1^) were taken from the data sheet of the glass manufacturer [26]. The simulations were performed for a range of core dimensions (core width varied from 1 to 2 μm, keeping the length to width ratio fixed at 1.5) and air gaps (varied from 0 to 500 nm).

**Figure 5:**
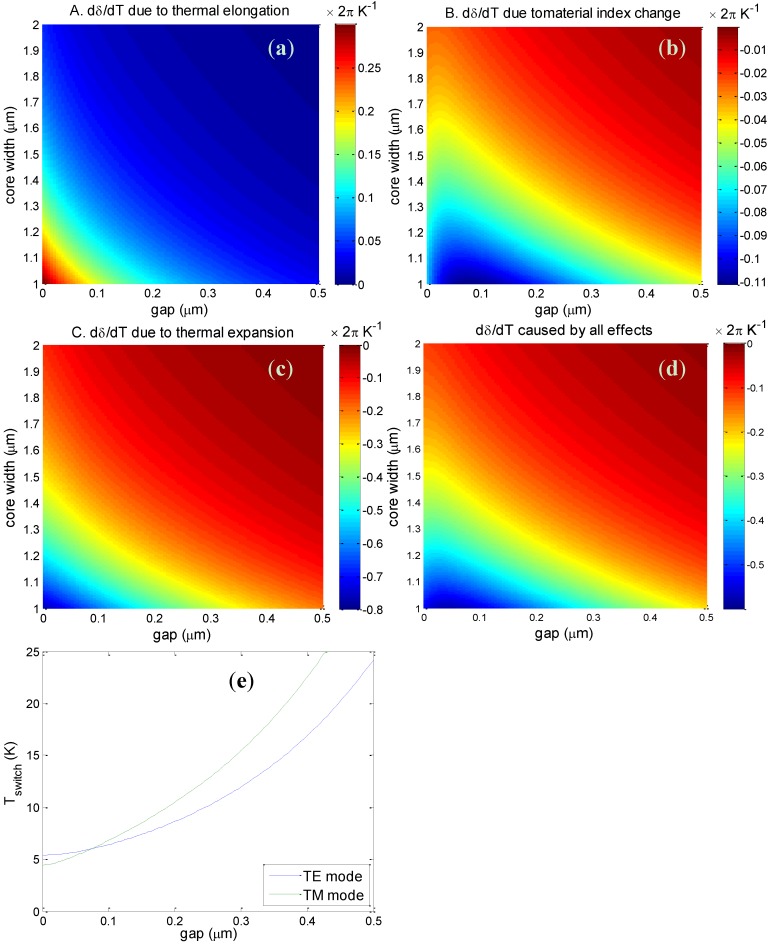
(**a**–**d**) Variation of the phase difference *d*δ/*dT* for TM polarization with core size and air gap size in a 50 cm piece of fiber induced by thermal effects A, B and C, and by the sum of these effects; (**e**) Temperature required to observe an optical switch in a 50-cm piece of fiber with core size 2 μm × 3 μm.

For this range of fiber dimensions the two cores are strongly coupled and the beat length was found to vary from a few tens of μm (for small cores and small gap) up to 1 mm (for large cores and large gap). The change of the phase difference *d*δ/*dT* between the TM supermodes induced by each of the three effects separately in a 50-cm long piece of fiber is shown in Figure 5a–c, while Figure 5d shows the total phase difference change caused by all of the effects. Similar results were also obtained for the TE supermodes. The figures show that the fiber sensitivity to temperature changes strongly depends on the core size and the air gap between the cores. Smaller cores and smaller core separations lead to stronger optical coupling between the cores and to larger sensitivity to temperature variations. As discussed above, effect A leads to an increase of δ with increasing temperature, while effects B and C decrease δ and the total phase difference change has a negative value. The contribution of the thermal expansion in the transverse direction (effect C) is approximately 2.5 times larger than the impact of the thermal elongation (effect A) and almost an order of magnitude larger than the effect of the material index change (effect B).

Calculations of the temperature of the cores required for switching the light from one core to the other for the TM and TE modes in a 50-cm long piece of fiber with a core size of 2 μm × 3 μm are shown in Figure 5e. The switching temperature varies from 5 to 25 K depending on the air gap between cores. This is of the same order of magnitude as the experimental switching temperature, but slightly larger. Note that following Equation (2) the phase change scales linearly with the fiber length and thus the required temperature change for optical switching is inversely proportional to the fiber length.

## 5. Discussion and Conclusions

In this work we reported the successful fabrication of a new kind of multi-material MOF based on lead silicate glass with integrated tin electrodes by a co-draw technique. The fiber has a dual-core structure, with two elliptical cores being independently suspended by thin membranes. The optical coupling between the two cores is extremely sensitive to any structural changes, and thus the fiber can be used as a sensor or an integrated all-fiber switch. The embedded conducting electrodes allow for novel electric actuation mechanisms operating at low voltages and/or low power due to their close vicinity to the optically guiding cores.

In previous work, we discussed two different methods of dual-core fiber actuation, mechanical and electrostatic. In the mechanical method one suspended core was moved by 8 nm by applying air pressure of ~100 mbar to one of the air holes through a slit in the cladding [17] leading to one optical switching cycle in a 40-cm long fiber. However, the mechanical method is slow and requires placing the fiber in a bulky pressure chamber. Alternatively, electrostatic MEMS-type actuation by induced electric dipole forces was proposed to allow for fast switching on a microsecond time scale [18]. This method relies on applying electric voltages to electrodes embedded in the fiber jacket, but because of the weak induced forces at realistic voltages the method also requires very thin and long membranes (e.g., 0.1 μm × 25 μm) supporting the fiber cores, making this an extremely challenging fiber design for fabrication. The fibers presented in this paper exhibit the required metal electrodes close to the cores for this actuation mechanism, but the supporting membranes are too stiff given the dimensions of Table 1. Our simulations suggest that voltages higher than ±200 V would have to be applied to the electrodes to observe optical switching in a 50-cm long fiber, generating an electric field sufficiently strong to lead to dielectric breakdown of air in the fiber holes [27].

Here we demonstrated another electrically induced actuation method of the dual-core fiber based on Joule heating: by passing electric current through the embedded metal electrodes, the temperature of the fiber increases slightly, changing the coupling length between the fiber cores. Optical switching in ~70 cm of fiber was observed by applying ~0.4 W of electric power. In a reference experiment using an external heater we found that a temperature difference of 5.6 °C is required to produce one optical switch. Numerical simulations further confirm the thermal origin of the actuation mechanism. The calculations suggest that in the range of the studied fiber dimensions the effect of thermal expansion in the transverse directions dominates over the change of material index with temperature and elongation of the fiber. This novel actuation mechanism has the advantage of working at relatively robust fiber geometries with thick membranes supporting the dual cores, but it requires Watt-level actuation powers and the switching time will be limited to the millisecond range by the thermal response time of the fibers. Further improvements of the fabrication technique will therefore be required to demonstrate dual-core optical fibers with longer and more flexible membranes for fast and low-power optical switching by electrostatic actuation.
